# Diffusive and Metabolic Constraints to Photosynthesis in Quinoa during Drought and Salt Stress

**DOI:** 10.3390/plants6040049

**Published:** 2017-10-17

**Authors:** Dilek Killi, Matthew Haworth

**Affiliations:** 1 Department of Agrifood Production and Environmental Sciences (DiSPAA), University of Florence, Piazzale delle Cascine 28, 50144 Florence, Italy; 2 The National Research Council of Italy, Tree and Timber Institute (CNR-IVALSA), Presso Area di Ricerca CNR, Via Madonna del Piano 10, Sesto Fiorentino, 50019 Florence, Italy; haworth@ivalsa.cnr.it

**Keywords:** *Chenopodium quinoa*, salinity, novel food crops, food security, stomatal conductance, mesophyll conductance, chlorophyll fluorescence

## Abstract

Quinoa (*Chenopodium quinoa* Willd.) has been proposed as a hardy alternative to traditional grain crops in areas with warm-to-hot climates that are likely to experience increased drought and salt stress in the future. We characterised the diffusive and metabolic limitations to photosynthesis in quinoa exposed to drought and salt stress in isolation and combination. Drought-induced pronounced stomatal and mesophyll limitations to CO_2_ transport, but quinoa retained photosynthetic capacity and photosystem II (PSII) performance. Saline water (300 mmol NaCl-equivalent to 60% of the salinity of sea-water) supplied in identical volumes to the irrigation received by the control and drought treatments induced similar reductions in stomatal and mesophyll conductance, but also reduced carboxylation of ribulose-1,5-bisphosphate carboxylase/oxygenase, regeneration of ribulose-1,5-bisphosphate, increased non-photochemical dissipation of energy as heat and impaired PSII electron transport. This suggests that ion toxicity reduced *P*_N_ via interference with photosynthetic enzymes and degradation of pigment–protein complexes within the thylakoid membranes. The results of this study demonstrate that the photosynthetic physiology of quinoa is resistant to the effects of drought, but quinoa may not be a suitable crop for areas subject to strong salt stress or irrigation with a concentration of saline water equivalent to a 300 mmol NaCl solution.

## 1. Introduction

Food security is jeopardized by climate change, population growth, unsustainable agriculture and the loss of agricultural land to urbanization/industrialization [1]. The development of crops with enhanced productivity and tolerance to abiotic stress may contribute toward sufficient future food production [2,3]. Quinoa (*Chenopodium quinoa* Willd.) has attracted attention as a novel food crop capable of producing comparatively large volumes of grain under adverse growth conditions [4,5]. Drought and salinity are two of the major causes of reduced plant growth and yield in areas with warm-to-arid climates [2,6], where quinoa has been proposed as an alternative to more traditional crops such as wheat (*Triticum aestivum*) [7]. Characterization of the diffusive and metabolic components of photosynthesis (*P*_N_) in quinoa exposed to drought and salt stress may provide insights into the suitability of quinoa as a crop species for cultivation in areas subject to these stresses.

As soil dries and the availability of water for uptake by plants declines, hydraulic and chemical signals induce stomatal closure to reduce transpirative water-loss. Decreased stomatal conductance (*G*_s_) results in increased diffusive resistance to CO_2_-uptake, reducing the availability of CO_2_ within the chloroplast envelope where *P*_N_ occurs [8]. As *P*_N_ decreases, less energy is utilized for photochemistry and an increasing amount is dissipated as heat via xanthophylls (non-photochemical quenching) [9]. If drought stress is severe or occurs over a prolonged period, plants may not be able to dissipate excess energy safely and the protective mechanisms that neutralize reactive oxygen species may become degraded [2]. This may result in impaired performance of photosystem II (PSII) [10,11] and a reduction in photosynthetic capacity [12]. Plants may also respond to drought by reducing the expression of genes encoding photosynthetic enzymes, leading to decreased photosynthetic capacity [13]. Field trials of quinoa in the Mediterranean found that the yield of plants grown under rain-fed conditions were 42.6% lower than those receiving irrigation equivalent to potential evapotranspiration [7]. The retention of yield under drought in quinoa may be related to increased concentrations of the drought stress hormone abscisic acid within the xylem [14] inducing stomatal closure and increasing water use efficiency (WUE) [15,16,17,18]. The maintenance of *P*_N_ in quinoa during drought stress may be associated with enhanced antioxidant activities minimizing oxidative stress [19].

Salt stress lowers soil water potential, resulting in reduced water availability for plant growth [20]. This induces stomatal closure and limits CO_2_-uptake in a manner similar to the effects of drought stress [2]. Salinity may also induce ion toxicity where Na^+^ inhibits metabolic function, in particular, enzymes involved in photosynthesis [21]. Irrigation of quinoa with increasingly saline water in Turkey (to an electrical conductivity of 40 dS m^−1^) did not significantly depress grain yield per plant [7]. Exposure to salt stress does result in a reduction in *P*_N_ and *G*_s_ in quinoa [22]. However, the deleterious effects of salt stress are less apparent in quinoa than in less salt-tolerant grain crops such as wheat (e.g., [23]) or rice (*Oryza sativa*) (e.g., [24]). The apparent tolerance of quinoa to salt stress may be related to its capacity to exclude Na^+^, increase the concentration of osmolytes such as K^+^ within leaves and reduce the number of stomata over the leaf surface to reduce maximum *G*_s_ [25,26,27].

Under field conditions, plants often experience drought and salt stress at the same time resulting in severely impaired growth [2]. However, the impact of these stresses in combination may not be additive in quinoa [28]. An in-depth analysis of the diffusive and biochemical components of the photosynthetic response to salinity and drought in combination and isolation would contribute to our understanding of the reported tolerance to abiotic stress in quinoa [7,28,29]. Moreover, the identification of attributes conducive to drought and salt tolerance may assist in the development of improved hardiness in new varieties of quinoa and/or staple crops that are more vulnerable to abiotic stress such as rice (e.g., [30]). We hypothesize that quinoa will maintain photosynthetic capacity and PSII performance under drought and salt stress, and that the limitations to *P*_N_ will be largely diffusive. This study aims to: (i) characterize the diffusive and biochemical constraints to *P*_N_ in quinoa subject to drought and/or salt stress; (ii) assess the performance of PSII electron transport and heat dissipation during drought and salt stress, and; (iii) discuss the photosynthetic response of quinoa to stress conditions likely to occur during growth in Mediterranean areas, and the potential to develop enhanced yield and tolerance of abiotic stress in quinoa through modification of photosynthetic and stomatal physiology.

## 2. Results

**Figure 1:**
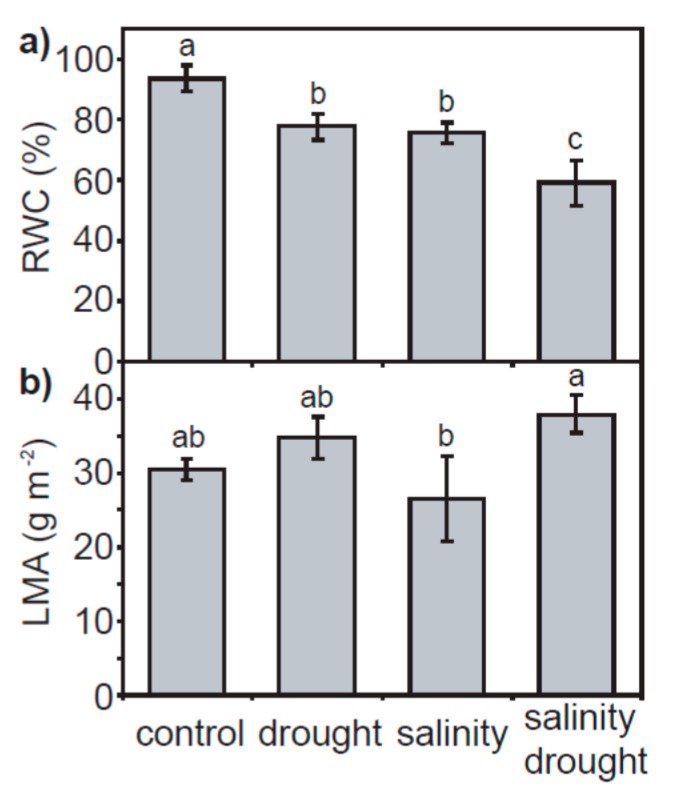
Relative water content (**a**) and leaf mass per area (**b**) of quinoa leaves from plants subject to the control, drought, salinity and salinity–drought treatments. Error bars indicate one standard deviation either side of the mean. Letters indicate significant difference using a one-way ANOVA and a least significant difference (LSD) post-hoc test.

**Figure 2:**
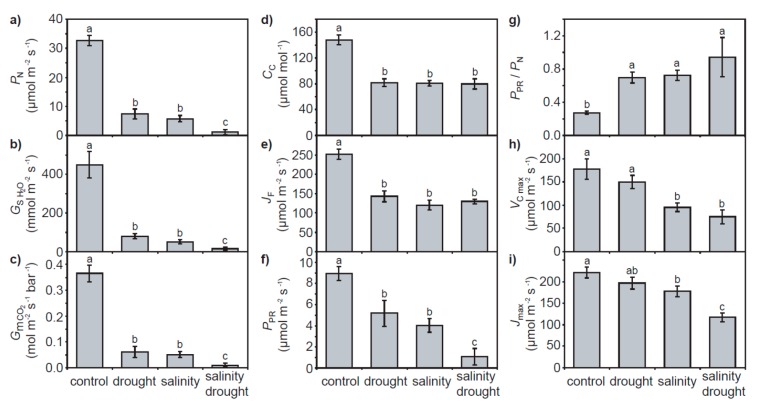
Photosynthetic parameters of quinoa subject to the control, drought, salinity and salinity–drought treatments: (**a**) photosynthesis; (**b**) stomatal conductance to water vapor; (**c**) mesophyll conductance to CO_2_; (**d**) concentration of [CO_2_] within the chloroplast envelope; (**e**) electron transport; (**f**) photorespiration; (**g**) ratio of photorespiration to photosynthesis; (**h**) the maximum carboxylation rate of RubisCO, and; (**i**) the maximum rate of electron transport for regeneration of RuBP. Error bars indicate one standard deviation either side of the mean. Letters indicate significant difference using a one-way ANOVA and an LSD post hoc test.

**Figure 3:**
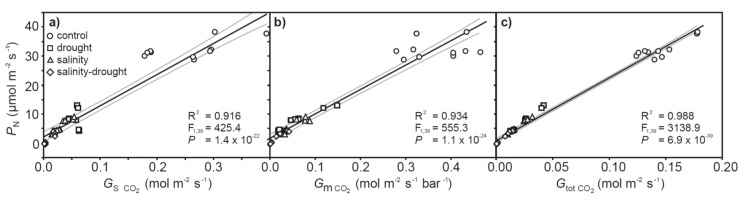
The relationship between photosynthesis with stomatal (**a**), mesophyll (**b**) and total (**c**) conductance to CO_2_. The black line indicates the line of best fit and the two grey lines either side indicate the 95% confidence intervals of the mean. Linear regression was used to assess the significance of any relationship.

Drought and salt stress resulted in significant respective 17.0 and 19.3% reductions in leaf relative water content (RWC). A combination of drought and salinity induced a greater 36.9% decrease in foliar RWC (Figure 1a). The leaf mass per area (LMA) of quinoa leaves was not significantly affected by the drought and salinity treatments with respect to control values; however, the LMA of leaves exposed to drought and salinity was significantly higher than those subjected to salinity only (Figure 1b). Drought and salinity in isolation induced respective reductions of 77.3% and 82.4% in leaf level *P*_N_. A combination of drought and salinity resulted in the lowest rates of *P*_N_ (96.5% lower than control values) (Figure 2a). These reductions in *P*_N_ with salinity and drought were associated with corresponding reductions in stomatal (Figure 2b) and mesophyll (Figure 2c) conductance. However, the reduction in *G*_s_ and mesophyll conductance (*G*_m_) values between the individual drought and salinity treatments to the combined salinity and drought treatment did not correspond to a lower concentration of [CO_2_] within the chloroplast envelope (*C*_c_) (Figure 2d); indeed, *C*_c_ was identical in all the salinity and/or drought treatments, approximately 45% lower than the levels of *C*_c_ in the control plants. This may be associated with the similarity in PSII electron transport rate (*J*_F_) values observed in the drought and salinity treatments (Figure 2e) used in the variable J calculation. Rates of *P*_N_ were positively correlated to stomatal, mesophyll and total conductance to CO_2_, with the significance of the relationship greatest between *P*_N_ and total conductance to CO_2_ *(G*_tot_) (Figure 3). Rates of photorespiration (*P*_PR_) declined respectively by 42.1% and 55.0% in the individual drought and salinity treatments, and were 87.9% lower in the combined drought and salinity treatment (Figure 2f). However, as a proportion of *P*_N_, *P*_PR_ was significantly greater in the plants exposed to drought and/or salt stress than the control (Figure 2g).

**Figure 4:**
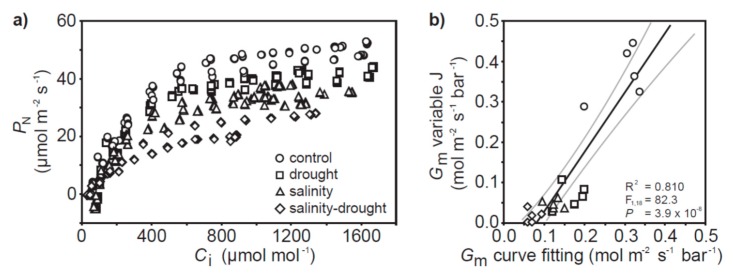
(**a**) *P*_N_-*C*_i_ response curves of quinoa subject to the control, drought, salinity and salinity–drought treatments; (**b**) comparison of *G*_m_ values calculated using the variable J [32] and *P*_N_-*C*_i_ curve fitting [31] methods. The black line indicates the line of best fit and the two grey lines either side indicate the 95% confidence intervals of the mean. Linear regression was used to assess the significance of any relationship.

The photosynthetic capacity of the quinoa plants was not significantly affected by the drought treatment (Figure 2h,j and Figure 4a). Salinity resulted in a lower maximum carboxylation rate of ribulose-1,5-bisphosphate carboxylase/oxygenase (RubisCO) (*V*c_max_) and lower maximum rate of electron transport for regeneration of ribulose-1,5-bisphosphate (RuBP)(*J*_max_), and a combination of drought and salinity further reduced *J*_max_ (Figure 2h,i). The *J*_max_/*V*c_max_ ratio was not significantly altered in any of the drought and/or salinity treatments with respect to the control plants (one-way ANOVA with LSD post hoc: F_3,17_ = 1.453; *p* = 0.270). Mesophyll conductance values calculated from the *P*_N_-*C*_i_ response curves [31] were broadly consistent with those determined by the variable J approach [32] (Figure 4b).

**Figure 5:**
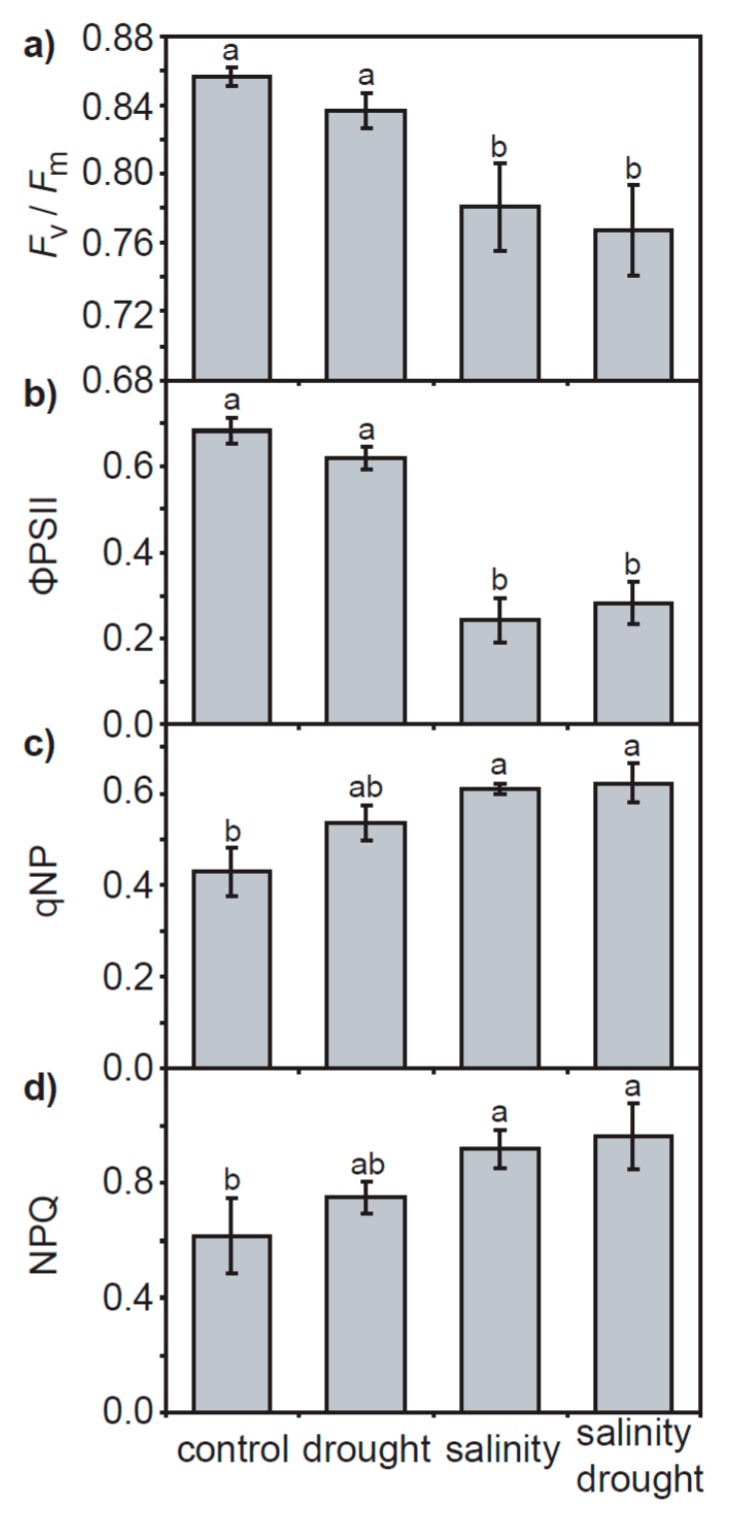
Pulse amplitude modulation chlorophyll fluorescence analysis of quinoa plants grown in the control, drought, salinity and salinity–drought treatments: (**a**) *F*_v_/*F*_m_; (**b**) ΦPSII; (**c**) qNP, and; (**d**) NPQ. Error bars indicate one standard deviation either side of the mean. Letters indicate significant difference using a Kruskal–Wallis non-parametric test.

**Figure 6:**
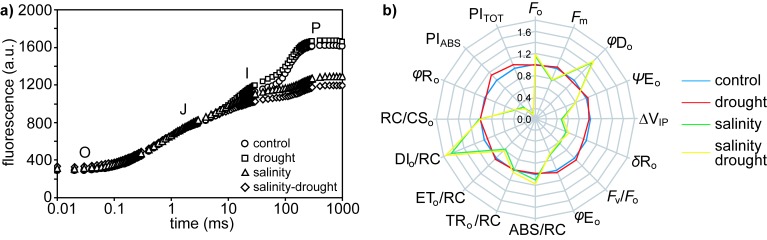
Analysis of the chlorophyll fluorescence transient of quinoa plants grown in the control, drought, salinity and salinity–drought treatments: (**a**) average OJIP induction curves; (**b**) spider plot of parameters (see supplementary information Table S1 for definitions and descriptions) extrapolated from the OJIP transient expressed in relation to control values.

Dark-adapted chlorophyll fluorescence parameters measured by pulse amplitude modulated fluorimetry indicated that the maximum (*F*_v_/*F*_m_: Figure 5a) and actual (ΦPSII: Figure 5b) quantum efficiencies of PSII and non-photochemical quenching of chlorophyll a fluorescence (qNP and NPQ: Figure 5c,d) were significantly lower in the salinity and salinity–drought treatments, while plants grown under the drought treatment exhibited no significant effect on PSII. Likewise, steps of chlorophyll fluorescence induction transient O-J-I-P (OJIP) curve analysis of the transient response of chlorophyll a fluorescence showed no significant effect of the drought treatment on PSII in quinoa (Figure 6 and Table 1). Salt stress in the salinity and salinity–drought treatments resulted in significant effects on the OJIP curves of the quinoa leaves: minimum fluorescence yield in dark-adapted conditions (*F*_o_), quantum yield of energy dissipation (*φ*D_o_), absorption of chlorophyll antennae per reaction centre (ABS/RC) and the flux of energy dissipated for each reaction centre (DI_o_/RC) were all significantly increased, while maximum fluorescence yield in dark-adapted conditions (*F*_m_), the efficiency of the electron chain flux in the I to P phase of the chlorophyll a fluorescence (ΔV_IP_), efficiency of electron carriers in reducing end electron acceptors at the PSI acceptor (*δ*R_o_), maximum quantum yield of PSII photochemistry (*F*_v_/*F*_m_), initial quantum yield of electron transport (*φ*E_o_), the electron flux beyond plastoquinone A per reaction centre (ET_o_/RC), a performance index based on the photochemical and non-photochemical energy absorption of chlorophyll antennae (PI_ABS_) and a performance index incorporating the concentration of reaction centres (PI_TOT_) were all reduced (Figure 6b and Table 1).

## 3. Discussion

The effects of physical and osmotic water deficit associated with drought and salinity on plant gas exchange and photosynthetic physiology can be broadly similar, particularly in the early stages of stress (e.g., [33,34]). In the present study, quinoa exhibited largely distinct photosynthetic and gas exchange responses to drought and salt stress. The effects of drought on *P*_N_ in quinoa were largely diffusive, characterized by reduced stomatal (e.g., [35]) and mesophyll (e.g., [8,36]) conductance to CO_2_. The rapid stomatal closure of quinoa is characteristic of ‘isohydric’ behavior [37] to maintain leaf water potential and RWC [38]. As drought induced significant reductions in the foliar RWC of quinoa (Figure 1a), this would suggest that the stress was pronounced. Nevertheless, removal of stomatal limitations via the use of prolonged exposure to sub-ambient [CO_2_] [39] indicated that quinoa had retained photosynthetic capacity (Figure 4a). Likewise, chlorophyll fluorescence analysis suggested that drought stress had little significant impact upon PSII photochemistry or non-photochemical quenching (Figure 5 and Figure 6). This is consistent with previous observations of enhanced antioxidant activity in quinoa [19] stabilizing the thylakoid membranes within the chloroplast envelope where PSII occurs [40]. Constraints to the uptake of CO_2_ via reduced *G*_m_ in the drought and salinity treatments was a significant factor in the *P*_N_ of quinoa (Figure 3b). Mesophyll conductance to CO_2_ is determined by physical [41,42] and biochemical [43] factors. The lack of any significant difference in LMA between the leaves of quinoa from the control and salinity and/or drought treatments would indicate that the reduced *G*_m_ observed in this study is largely the result of reduced biochemical uptake of CO_2_ across the mesophyll layer (e.g., [36]).

Exposure to salt stress in isolation led to reductions in transport of CO_2_ similar to those observed in drought-stressed quinoa (Figure 2c). Moreover, salinity also induced reductions in *V*c_max_ and *J*_max_ indicative of a loss of photosynthetic capacity (Figure 2h,i and Figure 4a). The reductions in *V*c_max_ found in salt-stressed quinoa would be consistent with impaired carboxylation of RubisCO [44,45] and/or reduced RubisCO content [46]. Salt stress also reduced the capacity for RuBP regeneration in quinoa indicative of reduced RuBP availability [45] and expression [47,48], particularly in the salinity drought treatment. The quinoa exposed to the salinity–drought treatment exhibited the lowest *G*_s_ of all the treatments (Figure 2b), suggesting that Na^+^ had not interfered with K^+^ stomatal signaling and guard cell osmoregulation (cf. [49]) consistent with exclusion of Na^+^ from salt-stressed quinoa [25].

Excessive concentrations of both Na^+^ and Cl^−^ interfere with plant physiology; however, Cl^−^ is generally more damaging to the function of photosynthetic enzymes and plant growth [50,51]. The impact of excess Cl^−^ is particularly evident in the performance of PSII [41], possibly due to disruption of the role of Cl^−^ in the oxygen-evolving complex of PSII [52,53]. Analysis of the chlorophyll fluorescence transient in quinoa exposed to the salinity and salinity–drought treatments would be consistent with ion toxicity effects on PSII function (Figure 5 and Figure 6). The reduction in *F*_v_/*F*_o_ (an indicator of the activity of the oxygen-evolving complex) values is consistent with reduced efficiency in the oxygen-evolving complex on the electron donor side of PSII [54]. The reduced maximum quantum efficiency of PSII (Figure 5a), enhanced dissipation of intercepted energy as heat (Figure 5c,d) and increased pool of reduced plastoquinone (the area above the OJIP curve) (Figure 6a) indicate reduced potential for photochemistry in the quinoa plants exposed to salinity [55]. The reduction in *F*_m_ values observed in the quinoa plants subject to salt stress may be suggestive of reduced numbers of light harvesting complex antennae [56] as the chlorophyll content declined due to Cl^−^ toxicity [57]. The reduction of the J, I and P levels of the fluorescence transient also indicates impaired electron transport [55] alongside the degradation of chlorophyll within the thylakoid complexes of salt-stressed quinoa (Figure 6a). The decrease in values of ΨE_o_, ∆V_IP_, *φ*R_o_ and *δ*R_o_ are consistent with reduced electron transfer from plastoquinone A to B and impaired PSI electron acceptors [58,59]. Salinity also reduced the number of open reaction centres in PSII (higher *F*_o_ indicating an increase in the number of inactive reaction centres incapable of electron transfer from reduced plastoquinone A), compounding the lower electron flux (i.e., reduced ET_o_/RC). This resulted in greater absorption (i.e., higher ABS/RC) and dissipation (i.e., higher DI_o_/RC) of energy per reaction centre (and reduced quantum yield of energy dissipation, *φ*D_o_), contributing to the lower overall performance indicators of PSII in the conservation and transport efficiency of excited electrons to PSI acceptors (i.e., lower PI_ABS_ and PI_TOT_) [55]. The disruption to the intersystem electron transport and end electron receptors in PSII is a major source of reactive oxygen species production [60], suggesting that the quinoa exposed to salinity experienced a higher level of oxidative stress than their control or drought treatment counterparts. The impact of salt stress on the performance of PSII in quinoa was mostly identical in the salinity and salinity–drought treatments (Figure 5 and Figure 6; Table 1), possibly suggesting that these effects were largely due to ion toxicity and not reduced water availability. 

## 4. Materials and Methods

### 4.1. Plant Material and Growth Conditions

Seeds of quinoa (*Chenopodium quinoa* Willd. var red head) were germinated in moist sand. Two weeks after germination, the plants were transplanted into 20 l pots filled with a 90% sand to 10% w/w commercial compost mixture (COMPO Terriccio Universale, COMPO Italia, Cesano Maderno, Italy) which has 22% of field capacity (FC) water content. The plants were grown outside from June to July 2016 in full sunlight in Sesto Fiorentino, Central Italy, and watered daily to pot capacity and supplied with a complete commercial nutrient solution twice a week to provide nutrients at free access rates (COMPO Concime Universale, NPK 7-5-7, B, Cu, Fe, Mn, Mo, Zn). After a further four weeks, when the plants were approximately 1 m high, the experimental treatments were instigated. The field capacity (FC) water content was determined gravimetrically [61]. The pots were weighed each day and the amount of water lost replaced for the control (80% of FC) and drought (30% of FC) water target. The treatments were: control (irrigation to 80% FC), drought (30% of FC), salinity (80% FC using 300 mmol saline water) and salinity–drought (30% FC using 300 mmol saline water). The saline water was prepared from a solution of deionized water and NaCl (≥99.5% purity; Sigma Aldrich, St. Louis, MO, USA) to a concentration of 300 mmol (i.e., 60% of the salinity of sea water). A concentration of 300 mmol was chosen as [62] observed that this was the concentration at which the effects of salinity became apparent in the seed germination and above–below ground growth parameters of quinoa. The drought and salinity–drought treatments were achieved by allowing the soil to dry to the target pot weight (this took two days) and then replenishing the water or saline solution each day to maintain the target weight. Five replicate plants were subject to each treatment.

### 4.2. Leaf Gas Exchange Analysis

Point measurements of leaf gas exchange and chlorophyll fluorescence were repeated on the uppermost fully expanded leaf of each replicate plant over the final two days of the two-week experimental period between 09:00 and 11:00 using a LiCor Li6400XT fitted with a 6400-40 2 cm^2^ leaf cuvette (Li-Cor, Inc., Lincoln NE, USA). Conditions in the leaf cuvette were set to a photosynthetic photon flux density (PPFD) of 2000 μmol m^−2^ s^−1^, leaf temperature of 25 °C, [CO_2_] of 400 μmol mol^−1^ and relative humidity of 60%. The multi-phase fluorescence setting was used with an initial saturating pulse of 8000 μmol m^−2^ s^−1^ [63]. Mesophyll conductance (*G*_m_) was determined using the variable J method described in [32]:

$$G_{m}=\frac{P_{N}}{C_{i}-\frac{\Gamma *\left[J_{F}+8\times \left(P_{N}+R_{d}\right)\right]}{J_{F}-4\times \left(P_{N}+R_{d}\right)}}$$

The CO_2_ compensation point to photorespiration (Γ*) was calculated using the RubisCO specificity factor described in [64]. The Kok method [65] was used to estimate respiration in the light (*R*_d_) (PPFD levels of 400, 300, 200, 100, 80, 60, 30, 0 μmol m^−2^ s^−1^). The effect of variation in the sub-stomatal concentration of [CO_2_] (*C*_i_) on *R*_d_ was corrected using the iterative method outlined in [66]. The PSII electron transport rate (*J*_F_) was calculated from chlorophyll fluorescence as:

$$J_{F}=PPFD\times \Phi PSII\times \alpha \times \beta$$

where the partitioning factor between photosystems I and II is considered to be 0.5 (β); leaf absorbance (α) is assumed to be 0.85 [67]; and, the actual quantum efficiency of PSII (ΦPSII) determined as:

$$\Phi PSII=\frac{{F_{m}}^{\prime }-F_{s}}{{F_{m}}^{\prime }}$$

where *F*_m_′ is the maximal fluorescence and *F*_s_ is the steady-state fluorescence under light-adapted conditions [11]. The concentration of [CO_2_] within the chloroplast envelope was calculated using the value of *G*_m_ derived from the variable J method as:

$$C_{c}=C_{i}-\frac{P_{N}}{G_{m}}$$

where *C*_i_ is the concentration of [CO_2_] within the internal sub-stomatal air-space [68]. Photorespiration (*P*_PR_) was determined following Sharkey [69]:

$$P_{\mathrm{PR}}=\frac{P_{N}+R_{d}}{\frac{C_{c}}{\Gamma *}-1}$$

Total conductance to CO_2_ was calculated as:

$$G_{\mathrm{tot}}=\frac{G_{s}\times G_{m}}{G_{s}+G_{m}}$$

The response of *P*_N_ to increasing *C*_i_ was determined in the last four days of the experimental treatment using a LiCor Li6400-40 attached to a 6 cm^2^ LiCor 6400-02B leaf cuvette with red and blue LEDs. The uppermost fully expanded leaf on five replicate plants for each treatment was analyzed. The *P*_N_-*C*_i_ response of quinoa is sensitive to light intensity [70]; therefore, a saturating PPFD of 2000 μmol m^−2^ s^−1^ was used. The concentration of [CO_2_] within the leaf cuvette was lowered to 50 μmol mol^−1^ for 60 min to fully open stomata and remove any diffusive limitations to *P*_N_ before [CO_2_] was increased in stages every 3 to 4 min when *P*_N_ had stabilized ([CO_2_] steps: 50, 100, 200, 300, 400, 600, 800, 1000, 1200, 1400, 1600, 1800, 2000 μmol mol^−1^) [39]. Leaf temperature remained at 25 °C and relative humidity at 60% throughout the *P*_N_-*C*_i_ response curve. To correct for diffusion leaks during the *P*_N_-*C*_i_ curve, a dead leaf was placed within the leaf cuvette and the step changes in [CO_2_] repeated following [71]. The maximum carboxylation rate of RubisCO (*V*c_max_), the maximum rate of electron transport for regeneration of ribulose-1,5-bisphosphate (RuBP) (*J*_max_) and mesophyll conductance (*G*_m_) were calculated from the *P*_N_-*C*_i_ curve [31]. The parameters *V*c_max_ and *J*_max_ were calculated from the *P*_N_-*C*_i_ curve and not the *P*_N_-*C*_c_ curve as this would require the assumption that *G*_m_ is constant at all [CO_2_] levels, while evidence suggests that *G*_m_ may vary with [CO_2_] [43].

### 4.3. Chlorophyll Fluorescence 

Chlorophyll fluorescence analyses were performed over the last three days of the experimental treatments on the uppermost fully expanded leaf of each plant. Transient analysis of chlorophyll, a fluorescence was undertaken using a Hansatech Handy-PEA (plant efficiency analyzer) fluorimeter (Hansatech, King’s Lynn, UK). Leaves were dark-adapted for 30 min and then exposed to a saturating light pulse (intensity > 3000 μmol m^−2^ s^−1^, excitation light of 650 nm) [55]. This results in a polyphasic transient of chlorophyll fluorescence: O (20–50 µs), J (2 ms), I (30 ms) and P (peak). A summary of the theoretical basis and analysis of OJIP curves is given in Strasser et al. [55] and Kalaji et al. [10]. The OJIP curves were analyzed using Biolyzer 4 HP v.3 (Bioenergetics Laboratory, University of Geneva, Geneva, Switzerland). The parameters extrapolated from the OJIP curve and analyzed in this study are: *F*_o_, minimum fluorescence yield in dark-adapted conditions; *F*_m_, maximum fluorescence yield in dark-adapted conditions; *φ*D_o_, quantum yield of energy dissipation (*F*_o_/*F*_m_) at time 0; ΨE_o_, the probability that harvested excitation energy is utilized for electron transport to the primary plastoquinone A acceptor of PSII; *F*_v_/*F*_o_, an indicator of the activity of the oxygen-evolving complex on the donor side of PSII; ΔV_IP_, a relative measure of the I to P phase of the chlorophyll a fluorescence transient indicating the efficiency of the electron chain flux through photosystem I (PSI); *δ*R_o_, efficiency of electron carriers in reducing end electron acceptors at the PSI acceptor; *F*_v_/*F*_m_, maximum quantum yield of PSII photochemistry; *φ*E_o_ initial quantum yield of electron transport at time 0; ABS/RC, absorption of chlorophyll antennae per reaction centre; ET_o_/RC, the electron flux beyond plastoquinone A per reaction centre; TR_o_/RC, the flux of trapped energy per reaction centre leading to the reduction of plastoquinone A; DI_o_/RC, the flux of energy dissipated for each reaction centre; RC/CS_o_, the density of PSII plastoquinone A reducing reaction centres; *φ*R_o_, quantum yield of the reduction of final stage acceptors at the PSI stage; PI_ABS_, a performance index based on the photochemical and non-photochemical energy absorption of chlorophyll antennae; PI_TOT_, a performance index incorporating the concentration of reaction centres, the quantum yield of PSII photochemistry, capacity for uptake of electrons between PSII and PSI and the efficiency of electron transfer from reduced intersystem electron acceptors to the final stage PSI electron [55]. More detailed definitions, descriptions and formulae for the PEA parameters used in this study are given in supplementary information Table S1.

The maximum (*F*_v_/*F*_m_) and actual (ΦPSII) quantum yields of photosystem II and the non-photochemical quenching co-efficient, qNP and NPQ, were recorded using a Hansatech Pulse-Amplitude-Modulation FMS-2 fluorimeter. After 30 min dark adaptation, the leaves were exposed to a saturating pulse of 10,000 μmol m^−2^ s^−2^, then actinic light of 1000 μmol m^−2^ s^−1^ for a minimum of 10 min followed by a second saturating pulse. To gauge energy dissipation via non-photochemical quenching, the co-efficient qNP and NPQ were used:

$$qNP=\frac{F_{m}-{F_{m}}^{\prime }}{F_{m}-F_{o}}\;NPQ=\frac{F_{m}-{F_{m}}^{\prime }}{{F_{m}}^{\prime }}$$

### 4.4. Relative Water Content and Leaf Mass per Area

At the end of the two-week treatment, the uppermost fully expanded leaves that had been used for gas exchange and chlorophyll fluorescence analyses were destructively sampled. Their fresh weight (FW) was recorded and then digital images were taken using a Sony DSC-T99 14 megapixel camera (Sony, Tokyo, Japan). The leaves were sealed inside falcon tubes with their petiole submerged below deionized water and kept in darkness for 24 h. Excess water on the surface of the leaves was removed using paper towels and their turgid weight (TW) recorded. The leaves were then dried for at least 72 h at 80 °C, when their weight remained constant this was considered to represent their dry weight (DW). The relative water content (RWC) of the leaves was determined as:

$$RWC=\left(\frac{FW-DW}{TW-DW}\right)\times 100$$

Leaf area was determined from the digital images of the fresh leaves using the software program ImageJ (National Institutes of Health, Bethesda, MD, USA). The leaf mass per area (LMA) of the leaves was taken as their dry mass per unit fresh area.

### 4.5. Statistical Analyses

Statistical analyses were performed using SPSS 20 (IBM, Armonk, NY, USA). To test the effect of drought, salinity or salinity–drought on the normally distributed physiological and morphological parameters of quinoa we used a one-way ANOVA with an LSD post hoc test. Linear regression was used to investigate possible relationships between *P*_N_ to conductance to CO_2_. The Shapiro–Wilk test was applied to the *ChlF* data to assess the normality, and the Levene test was used to investigate homogeneity of variance. *ChlF* data was not normally distributed and the variance was not homogenous, and therefore the non-parametric Wilcoxon Signed-Rank Test was applied to the *ChlF* data to assess the mean rank differences of means between the control and groups subject to drought and/or salinity.

## 5. Conclusions

The results of this study have demonstrated that quinoa is a relatively drought-tolerant crop species exhibiting diffusive limitations to *P*_N_ under water deficit. The retention of photosynthetic capacity and PSII performance under drought indicate highly effective protective mechanisms to dissipate excess energy and neutralize oxidative stress. Stomatal and mesophyll limitations to the transport of CO_2_ played a major role in the response of quinoa to drought and salt stress. Modification of the physical and biochemical properties of the mesophyll layer may further enhance the photosynthetic performance of quinoa under drought stress (e.g., [72]). Salt stress induced short-term diffusive but also longer-term metabolic limitations to CO_2_ assimilation in quinoa. The deleterious effect of salinity was apparent in impaired RubisCO carboxylase activity, RuBP regeneration and PSII performance. Analysis of the chlorophyll fluorescence transient suggests that electron transport was impaired throughout PSII in salt-stressed quinoa. Salt stress degraded and damaged the pigment–protein complexes of the thylakoid membrane, likely inducing oxidative stress. While quinoa performed well under drought stress conditions, retaining photosynthetic capacity, it showed strong negative effects of salinity. This may suggest that quinoa would be suitable for cultivation in drought-prone Mediterranean areas not subject to strong salt stress (e.g., where irrigation is performed using sea-water: [73]). Selection of quinoa genotypes on the basis of gas exchange responses to salinity may be effective in identifying varieties capable of retaining photosynthetic function in growth conditions characterized by drought and salinity.

## Figures and Tables

**Table 1:** Analysis of parameters calculated from the chlorophyll fluorescence OJIP transient (Figure 6a) of quinoa plants under control, drought, salinity and a combination of salinity and drought treatments. Values are the means of five replicates (three measurements per replicate). ± indicates standard error. Values followed by different letters are significantly different at the *p* < 0.05 level using a Kruskal–Wallis nonparametric test.

**Treatment**	***F*_o_**	***F*_m_**	***φ*D_o_**	**ΨE_o_**
control	260 ± 10.0 ^b^	1621 ± 19.3 ^a^	0.180 ± 0.007 ^b^	0.665 ± 0.014 ^a^
drought	257 ± 4.2 ^b^	1666 ± 12.8 ^a^	0.172 ± 0.003 ^b^	0.686 ± 0.007 ^a^
salinity	297 ± 9.8 ^a^	1252 ± 26.7 ^b^	0.263 ± 0.012 ^a^	0.516 ± 0.011 ^b^
salinity–drought	306 ± 5.1 ^a^	1270 ± 33.6 ^b^	0.268 ± 0.007 ^a^	0.514 ± 0.018 ^b^
**Treatment**	**ΔV_IP_**	***δ*R_o_**	***F*_v_/*F*_o_**	***φ*E_o_**
control	0.390 ± 0.014 ^a^	0.587 ± 0.015 ^a^	4.64 ± 0.18 ^a^	0.546 ± 0.015 ^a^
drought	0.380 ± 0.010 ^a^	0.554 ± 0.013 ^a^	4.84 ± 0.10 ^a^	0.568 ± 0.007 ^a^
salinity	0.184 ± 0.010 ^b^	0.356 ± 0.016 ^b^	2.89 ± 0.17 ^b^	0.381 ± 0.012 ^b^
salinity–drought	0.197 ± 0.010 ^b^	0.383 ± 0.015 ^b^	2.75 ± 0.11 ^b^	0.376 ± 0.015 ^b^
**Treatment**	**ABS/RC**	**TR_o_/RC**	**ET_o_/RC**	**DI_o_/RC**
control	1.574 ± 0.024 ^b^	1.291 ± 0.021 ^ab^	0.857 ± 0.020 ^a^	0.283 ± 0.013 ^b^
drought	1.545 ± 0.015 ^b^	1.280 ± 0.010 ^ab^	0.877 ± 0.010 ^a^	0.266 ± 0.006 ^b^
salinity	1.736 ± 0.069 ^ab^	1.270 ± 0.030 ^b^	0.655 ± 0.020 ^b^	0.466 ± 0.041 ^a^
salinity–drought	1.846 ± 0.051 ^a^	1.348 ± 0.030 ^a^	0.693 ± 0.028 ^b^	0.498 ± 0.024 ^a^
**Treatment**	**RC/CS_o_**	***φ*R_o_**	**PI_ABS_**	**PI_TOT_**
control	184.6 ± 6.2 ^b^	0.320 ± 0.013 ^a^	61.5 ± 6.1 ^a^	30.6 ± 4.2 ^a^
drought	185.1 ± 2.7 ^ab^	0.314 ± 0.009 ^a^	69.6 ± 3.6 ^a^	32.7 ± 2.7 ^a^
salinity	188.8 ± 2.8 ^a^	0.135 ± 0.007 ^b^	19.0 ± 2.1 ^b^	3.1 ± 0.5 ^b^
salinity–drought	184.6 ± 4.0 ^ab^	0.143 ± 0.008 ^b^	16.4 ± 1.8 ^b^	2.8 ± 0.4 ^b^

## Supplementary Materials

The following are available online at www.mdpi.com/2223-7747/6/4/49/s1, Table S1: Diffusive and metabolic constraints to photosynthesis in quinoa during drought and salt stress.

## Abbreviations

*P*_N_	photosynthesis
*P*_PR_	photorespiration
*G*_s_	stomatal conductance
*G*_m_	mesophyll conductance
*G*_tot_	total conductance to CO_2_
*C*_i_	concentration of [CO_2_] within the internal sub-stomatal air-space
*C*_c_	concentration of [CO_2_] within the chloroplast envelope
RuBP	ribulose-1,5-bisphosphate
RubisCO	ribulose-1,5-bisphosphate carboxylase/oxygenase
*V*_Cmax_	ribulose-1,5-bisphosphate; maximum carboxylation rate of RubisCO
*J*_max_	maximum rate of electron transport for regeneration of RuBP
Γ*	CO_2_ compensation point to photorespiration
*R*_d_	respiration in the light
RWC	relative water content
LMA	leaf mass per area
PSI	photosystem I
PSII	photosystem II
*J*_F_	PSII electron transport rate
*F*_o_	minimum fluorescence yield in dark-adapted conditions
*F*_v_/*F*_o_	an indicator of the activity of the oxygen-evolving complex on the donor side of PSII
*φ*D_o_	quantum yield of energy dissipation
ABS/RC	absorption of chlorophyll antennae per reaction centre
DI_o_/RC	flux of energy dissipated for each reaction centre
*F*_m_	maximum fluorescence yield in dark-adapted conditions
ΦPSII	actual fluorescence yield in light-adapted steady-state conditions
ΔV_IP_	efficiency of the electron chain flux in the I to P phase of the chlorophyll a fluorescence
*δ*R_o_	efficiency of electron carriers in reducing end electron acceptors at the PSI acceptor
*F*_v_/*F*_m_	maximum quantum yield of PSII photochemistry
*φ*E_o_	initial quantum yield of electron transport
ET_o_/RC	electron flux beyond plastoquinone A per reaction centre
PI_ABS_	a performance index based on the photochemical and non-photochemical energy absorption of chlorophyll antennae
PI_TOT_	performance index incorporating the concentration of reaction centres
*F*_m_’	maximal fluorescence under light-adapted conditions
*F*_s_	steady-state fluorescence under light-adapted conditions
ΨE_o_	probability that harvested excitation energy is utilized for electron transport to the primary plastoquinone A acceptor of PSII
TR_o_/RC	flux of trapped energy per reaction centre leading to the reduction of plastoquinone A
RC/CS_o_	density of PSII plastoquinone A reducing reaction centres
*φ*R_o_	quantum yield of the reduction of final stage acceptors at the PSI stage
*OJIP*	major phases of chlorophyll fluorescence induction transient rise from O to P (O-J-I-P)
*O*	20–50 μs (O-step)
*J*	2 ms (J-step)
*I*	30 ms (I-step)
*P*	around 0.8 s (P-step; peak) which indicates highes fluorescence intensity (Fm)
