# Supplementary material for: Diffusive and Metabolic Constraints to Photosynthesis in Quinoa during Drought and Salt Stress
Source: Plants (Basel). 2017 Oct 17;6(4):49. doi: 10.3390/plants6040049 (PMC5750625; doi:10.3390/plants6040049)
Supplement: Supplementary file 1 [file plants-06-00049-s001.pdf]

Supplementary Materials: Diffusive and Metabolic Constraints to Photosynthesis in Quinoa during Drought and Salt Stress

Dilek Killi and Matthew Haworth

Table S1: Chlorophyll *a* fluorescence parameters and formulas used in the fluorescence transient analysis.

Parameters	Formulas and Definition
F_o	Minimal fluorescence from a dark-adapted leaf
F_m	Maximal fluorescence from a dark-adapted leaf
F_v	Maximal variable fluorescence from a dark-adapted leaf. $F_v = F_m - F_o$
V_J	Relative variable fluorescence at 3 ms. $V_J = (F_{2\,ms} - F_o)/(F_m - F_o)$
V_I	Relative variable fluorescence at 30 ms. $V_I = (F_{30\,ms} - F_o) / (F_m - F_o)$
φP_o	$Fv/F_m = [F_m - F_o]/F_m = 1 - F_o/F_m$ = maximum quantum yield of PSII primary photochemistry (at t = 0), measured in samples in dark-adapted state. F_v/F_m expresses the probability that an absorbed photon will be trapped by the PSII reaction centre.
φE_o	$(1 - F_o/F_m)(1 - V_J)$ = Quantum yield of electron transport (at t = 0).
φR_o	$(1 - F_o/Fm)(1 - V_I)$ = Quantum yield for reduction of end electron acceptors at the PSI acceptor side.
φD_o	F_o/F_m = Quantum yield (at t = 0) of energy dissipation
ΨE_o	$1 - V_J = 1 - (F_{2\,ms} - F_o)/(F_m - F_o)$. Ψ_{Eo} expresses the probability that the energy of a trapped excitation is used for electron transport beyond plastoquinone-A.
δR_o	$(1 - V_I)/(1 - V_J)$ = Efficiency with which an electron from the intersystem electron carriers moves to reduce end electron acceptors at the PSI acceptor side.
$\Delta V_{I\text{–}P}$	$1 - V_I = (F_m - F_{30\,ms})/ (F_m - F_o)$, I–P phase. This parameter indicates relative contribution of the I-P phase to the fluorescence transient OJIP; it is regarded as a measure of the efficiency of electron flux through PSI to reduce the final acceptors of the electron transport chain, i.e., ferredoxin and NADP.
ABS/RC	$(1 - Chl_{RC}/Chl_{tot})/(Chl_{RC}/Chl_{tot})$ = Absorption flux (of antenna chlorophylls) per reaction centre (RC).
PI_{ABS}	$(RC/ABS)\times[\varphi_{Po}/(1 - \varphi_{Po})]\times[\Psi E_o/(1 - \Psi E_o)]$. Performance index on absorption bases; absorption of antenna Chls of PSII. This measure incorporates photochemical and non-photochemical processes, such as absorption and trapping of excitation energy, electron transport beyond the plastoquinone-A and dissipation of excess excitation energy.
PI_{TOT}	Performance Index total (PI_{TOT}) is the potential for energy conservation from photons absorbed by PSII to the reduction flux (RE) of PSI end acceptors. The Pi_{tot} is a multi-parametric indicator of four measures of photosynthetic electron transport: (1) the concentration of reaction centres; (2) the quantum yield of PSII photochemistry; (3) the capacity for uptake of electrons in the electron chain between PSII and PSI; (4) the efficiency with which an electron can transfer from the reduced intersystem electron acceptors to the PSI end electron [1,2].<br>$PI_{TOT} = PI_{ABS}\,[\delta_{Ro}/(1 - \delta_{Ro})]$<br>where $\delta_{Ro} = (1 - V_J)/(1 - V_J) = (F_m - F_I)/ (F_m - F_J)$.<br>$\delta_{Ro}$ is the efficiency of an electron can transport from a reduced PQ to PSI end electron acceptor.

**TR_o/RC**	$M_o(1/V_J)$ = Trapping flux (leading to plastoquinone-A reduction) per RC.
**ET_o/RC**	$M_o(1/V_J)\Psi_o$ = Electron transport flux (further than plastoquinone-A) per RC.
**RC/CS_o**	$F_o\ \varphi P_o\ (V_J/M_o)$ = Density of RCs (plastoquinone-A reducing PSII reaction centers).
**DI_o/RC**	$(ABS/RC - TR_o/RC)$ = Dissipated energy flux per RC.
**M_o**	Slope of the curve at the origin of the fluorescence rise. It is a measure of the rate of the primary photochemistry. $M_o = 4\times(F_{300\,\mu s} - F_o)/(F_m - F_o)$
**ChlF steps**	The *ChlF* induction phase has different time steps called as: 20–50 μs (O-step), 2 ms (J-step), 30 ms (I-step), around 0.8 s (P-step; peak) and generally denoted F_o, F_J and F_I. The last step (P-step) indicates the highest fluorescence intensity (F_m), when saturating light is used.

References

1. Strasser, R.J.; Tsimilli-Michael, M.; Srivastava, A. Analysis of the chlorophyll a fluorescence transient. In *Chlorophyll a Fluorescence*; Papageorgiou, G., Govindjee, F.C., Gantt, E., Golbeck, J., Golden, S., Eds.;. Springer: Dordrecht, The Netherlands, 2004; pp. 321–362.
2. Strasser, R.J.; Tsimilli-Michael, M.; Qiang S.; Goltsev, V. Simultaneous in vivo recording of prompt and delayed fluorescence and 820-nm reflection changes during drying and after rehydration of the resurrection plant Haberlea rhodopensis. *Biochim. Biophys. Acta (BBA) Bioenergy*. **2010**, *1797*, 1313–1326.
